# Loss of *lkb1* Expression Reduces the Latency of ErbB2-Mediated Mammary Gland Tumorigenesis, Promoting Changes in Metabolic Pathways

**DOI:** 10.1371/journal.pone.0056567

**Published:** 2013-02-22

**Authors:** Rafaela Andrade-Vieira, Zhaolin Xu, Patricia Colp, Paola A. Marignani

**Affiliations:** 1 Department of Biochemistry and Molecular Biology, Faculty of Medicine, Dalhousie University, Halifax, Nova Scotia, Canada; 2 Department of Pathology, Faculty of Medicine, Dalhousie University, Halifax, Nova Scotia, Canada; Boston University School of Medicine, United States of America

## Abstract

The tumor suppressor kinase LKB1 is mutated in a broad range of cancers however, the role of LKB1 mammary gland tumorigenesis is not fully understood. Evaluation of human breast cancer tissue microarrays, indicate that 31% of HER2 positive samples lacked LKB1 expression. To expand on these observations, we crossed STK11*^fl/fl^* mice with mice genetically engineered to express activated Neu/HER2-MMTV-Cre (NIC) under the endogenous *Erbb2* promoter, to generate STK11*^−/−/^*NIC mice. In these mice, the loss of *lkb1* expression reduced the latency of ErbB2-mediated tumorigenesis compared to the latency of tumorigenesis in NIC mice alone. Analysis of STK11^−/−/^NIC mammary tumors revealed hyperactivation of mammalian target of rapamycin (mTOR) through both mTORC1 and mTORC2 pathways as determined by the phosphorylation status of ribosomal protein S6 and AKT. Furthermore, STK11^−/−/^NIC mammary tumors had elevated ATP levels along with changes in metabolic enzymes and metabolites. The treatment of primary mammary tumor cells with specific mTOR inhibitors AZD8055 and Torin1, that target both mTOR complexes, attenuated mTOR activity and decreased expression of glycolytic enzymes. Our findings underscore the existence of a molecular interplay between LKB1-AMPK-mTORC1 and ErbB2-AKT-mTORC2 pathways with mTOR at its epicenter, suggestive that loss of LKB1 expression may serve as a marker for hyperactivated mTOR in HER2 positive breast cancer and warranting further investigation into therapeutics that target LKB1-AMPK-mTOR and glycolytic pathways.

## Introduction

The tumor suppressor serine-threonine kinase LKB1, also referred to as STK11, is responsible for Peutz-Jeghers Syndrome (PJS), an autosomal-dominant disorder characterized by mucocutaneous hyper-pigmentation and benign gastrointestinal hamartomatous polyps and is attributed to mutations in LKB1 [1]. In PJS, the risk of breast cancer is the second highest after gastrointestinal cancers [2], while in non-PJS population, a correlation between loss of LKB1 expression in breast cancer, as determine by tissue microarray (TMA) and poor prognosis has been identified [3]. We recently discovered that LKB1 functions as a coactivator of estrogen receptor alpha (ERα) through direct binding with the hormone receptor [4]. In this study, we demonstrated for the first time a functional link between LKB1 and ERα, broadening the scientific scope of LKB1 and laying the groundwork for further investigations into the role of LKB1 in breast biology. For more detail about LKB1 signaling in disease please refer to the following review [5].

When in complex with pseudokinase STRAD and adaptor protein MO25, LKB1 catalytic activity is enhanced [6] allowing for the phosphorylation and activation of AMPK on threonine 172 [7], [8]. A link between the LKB1-AMPK pathway and disease pathways is the protein tuberin, the product of the tuberous sclerosis complex 2 gene (*TSC2*), that represses mammalian target of rapamycin (mTOR) activity when phosphorylated by AMPK on S1387 [9]. mTOR is a serine/threonine kinase that is the catalytic component of two distinct signaling pathways, mTOR complex 1 (mTORC1) and mTORC2 [10]. Both complexes are activated in response to growth factor signals mediated by the phosphoinositide 3-kinase (PI3K)/AKT signaling and the availability of nutrients to the cell [10]. A broad spectrum of cancers have been found to express aberrant mTOR signaling, whereby several pivotal proteins such as AMPK, AKT and S6 kinase (S6K) are functionally altered.

Previously, conditional hypomorphic LKB1*^fl/fl^* mice [11] were crossed with ovine beta-lactoglobulin gene (BLG)-Cre mice to excise *lkb1* from mammary glands of multiparous mice. In this model, loss of LKB1 expression gave rise to tumors on average by 16 months [12]. More recently Klefström and colleagues [13] analyzed the role of LKB1 and c-Myc in mammary gland development and tumorigenesis with a specific emphasis on the maintenance of epithelial integrity. Interestingly, the outcome of the Klefström study is in agreement with our earlier work [14] that describes how LKB1 catalytic deficient mutants gain oncogenic properties, driving the expression of oncogenes. How the loss of LKB1 expression leads to changes in downstream signaling pathways and how these pathways may be involved in mammary gland tumorigenesis require further investigation.

The ErbB family is implicated in mediating oncogenesis of epithelial-derived cancers and is reported to be overexpressed in approximately 20–30% of invasive breast cancers, more specifically in high grade ductal carcinoma *in situ* (DCIS) along with other oncogenes, cyclin D1 at 40–50% [15], and c-myc at 15–25% [16]. While 30–60% of breast cancer express mutations in the tumor suppressor p53 and approximately 10% express mutations in the polyposis syndrome tumor suppressor phosphatase and tensin homolog (PTEN), mutations in Neu/HER2 (ErbB2) are often found in conjunction with loss of function mutations in tumor suppressor proteins [17]. Interestingly, inactivating mutations in PTEN are associated with Herceptin resistance [18], [19].

To explore the interplay between Neu/HER2 and PTEN, conditional PTEN mice were interbred with constitutively active Neu/HER2 mice (MMTV-NIC) [20] (referred to herein as NIC mice), resulting in a dramatic increase in the rate of oncogenesis with corresponding amplification of the PI3K/AKT pathway but not mTOR signaling pathway [20].

## Materials and Methods

### Mice

All animal husbandry and studies were conducted in strict accordance with the Canadian Council on Animal Care. Protocol #10-009 was approved by the Committee on Laboratory Animals (UCLA), Dalhousie University. STK11*^fl/fl^* (129S6-*Stk11^tm1Rdp^*) [21] were from the NCI Mouse Repository, transgenic mice expressing oncogenic ErbB2 followed by an internal ribosomal entry sequence and a MMTV promoter driving Cre recombinase (generous gift from Dr. William Muller) [20]. STK11*^fl/fl^* mice were interbred with NIC mice to generate STK11*^−/−/^*NIC mice. All mice were palpated every three days to monitor for mammary tumors, grooming abnormalities and weight change.

### Histology

Whole mounts of mammary gland were stained using carmine (Sigma C1022) and aluminum potassium sulfate (Sigma A7167). Formalin fixed and paraffin embedded tissue were prepared for histology followed by hematoxylin and eosin (H/E) staining. Tissue sections were incubated in primary antibodies: LKB1 (Santa Cruz) 1∶30, Ki-67 (Santa Cruz) 1∶30, E-cadherin (Cell Signaling) 1∶100, pErbB2 1∶100 (Santa Cruz), and pS6 (S235/S236) 1∶100 (Cell Signaling). Human breast cancer TMAs were purchased from Biomax USA and prepared for IHC as per manufacturer’ recommendations. Scoring for all imaging was conducted in triplicate. Images were obtained using Nikon Eclipse TE 2000-E, mounted with a Q-Imaging CCD camera and acquired using the Simple PCI software as previously described [4].

### Metabolic Profile

Mammary tumors from three separate STK11^−/−/^NIC mice, and mammary glands from three separate WT mice were excised and analyzed for metabolites by NMR analysis (Chenomx, Edmonton, Alberta, Canada). Samples were prepared and analyzed as previously described [22]. Multivariate statistical analysis was performed using SIMCA P+ Version 12.0.1. ATP concentrations were measured from primary MECs using ATP Bioluminescence assay (Sigma).

### Kinase Inhibitors

The immunosuppressant and mTOR inhibitor Rapamycin was from Calbiochem, mTOR ATP-competitive inhibitors Torin1 was a generous gift from Drs. Sabatini and Grey [23], AZD8055 and BIBW2992 (Afatinib) were from Selleck, Chemicals. Treatments using mTOR inhibitors were based on previous drug studies conducted in the LKB1 conditional mice [24]–[26]. While treatments using the irreversible ErbB1/ErbB2 inhibitor BIBW2992 were based on studies conducted in conditional mouse model of lung cancer [27].

### Statistical Analysis

Results were derived from a minimum of three independent experiments. For Kaplan-Meir curves, Log ranked tests, scores of 3 or higher were considered significant (p<0.0001). ATP concentrations are mean values from 2 µg of total protein from MEC lysates ± SEM, p<0.01. For NMR data, metabolites were corrected for the weight of the tumor or normal mammary tissue, and reported as mean (µM/mg) ± SEM, p<0.05 or p<0.01.

## Results

### Loss of LKB1 Expression in High-grade Breast Cancer

**Figure 1:**
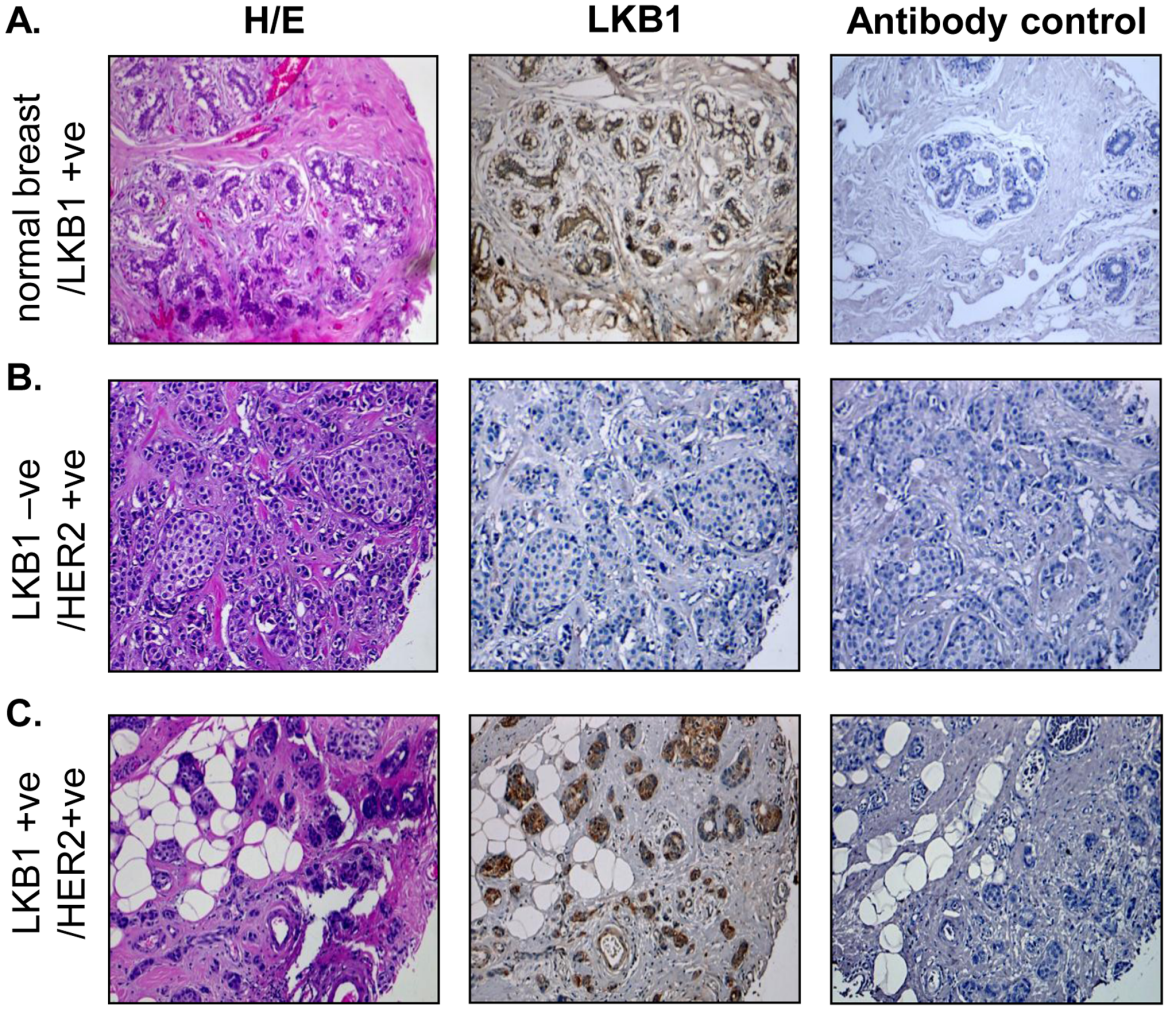
Loss of LKB1 expression in highgrade tumors. **A**, Human breast cancer tissue microarrays containing 120 duplicate core samples were analysed by IHC for LKB1 expression. H/E staining (left column), LKB1 expression (middle column), and antibody control (right column). **A,** LKB1 expression in normal mammary gland, **B,** loss of LKB1 expression/HER2 positive and **C,** LKB1 positive/HER2 positive.

We evaluated commercially prepared human breast cancer tissue microarrays (TMAs) (Table 1). Duplicate core samples (102) of high-grade tumors with corresponding HER2 expression status were scored for LKB1 expression by immunohistochemistry (IHC) (Table 1, Fig. 1). We found that 31% (32/102) tumors lacked LKB1 expression compared to tumors that expressed LKB1, p<0.05 (Table 1). We also observed that LKB1 expression was localized to luminal epithelium of normal breast tissues (Fig. 1A, C). LKB1 expression was scored as LKB1-ve (null) for no detectable LKB1 staining (Fig. 1B), LKB1+ (low), LKB1++ (medium) and LKB1+++ (high), compared to antibody controls (Supporting Information Fig.S1).

**Table 1:** LKB1 Expression in high grade breast cancer.

	HER2+ve			HER2−ve	
TMA Cores	LKB1−ve	LKB1+	LKB1+++	LKB1−ve	LKB1+ve
DCIS 8	1±0.3 (12.5%)	24±2.0 (26%)	11±1.5 (12%)	19±0.8 (20%)**	9±0.5 (9%)
DCIS 8	1±0.3 (12.5%)	2±0.3 (25%)	1±0.3 (12.5%)	3±1.1 (37.5%)**	1±0.3 (12.5%)

### Activated Neu/HER2 Enhances Onset of Tumorigenesis

**Figure 2:**
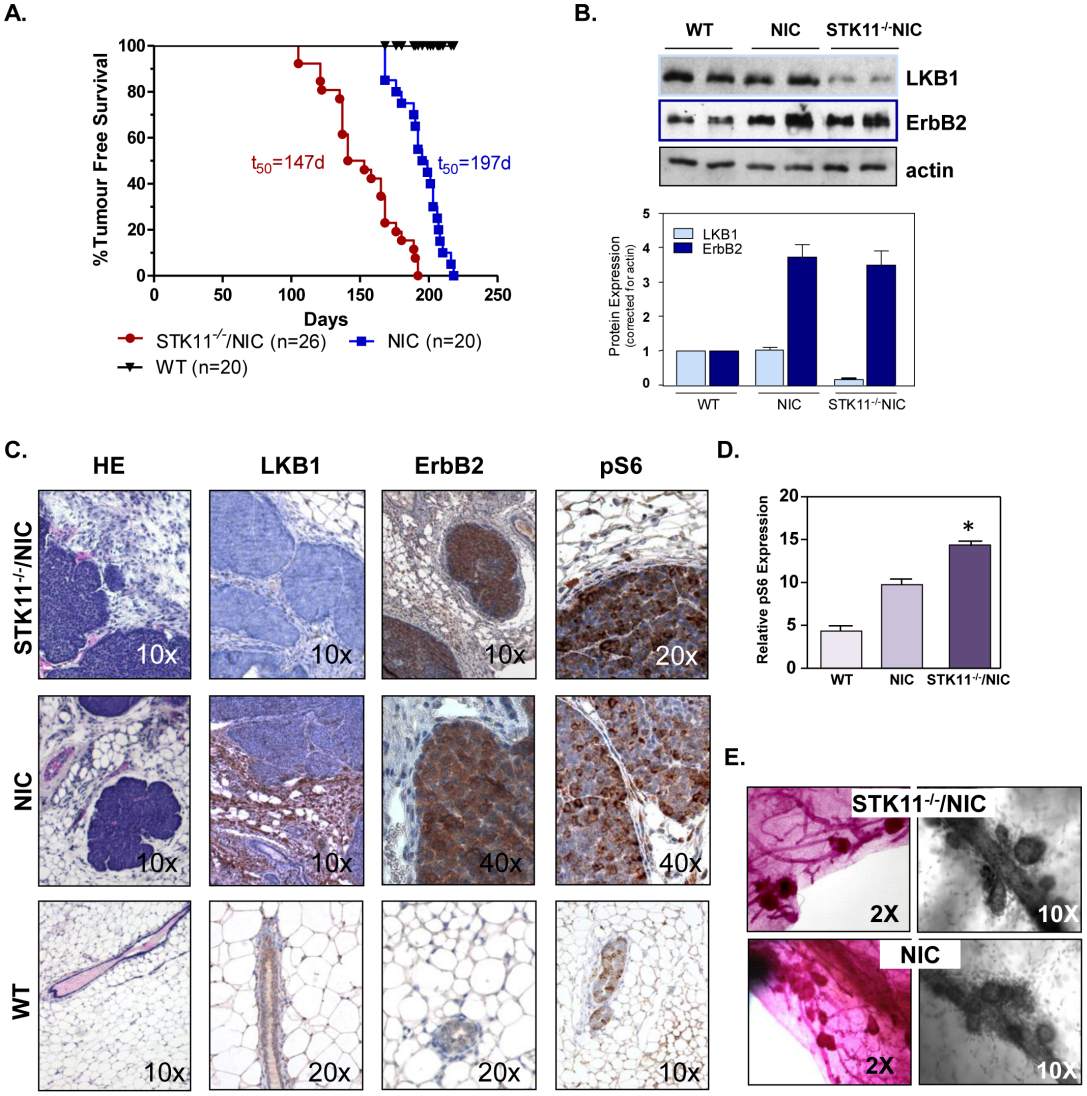
Loss of LKB1 expression reduces latency of ErbB2-mediated carcinogenesis. **A,** Kaplain-Meir percent tumor free survival curves for WT, STK11*^−/−/^*NIC, and NIC mice. Log Ranked Test, p<0.0001. T_50_ represents time in days where 50% of the females presented with tumors and n is the number of female mice analysed. **B,** Western blot analysis confirming expression of LKB1, ErbB2, and actin (loading control) using antibodies detailed in the Methods section. Graphic representation of protein expression corrected for actin ± SD. **C,** Tumors were evaluated for LKB1, ErbB2, and pS6 expression in STK^−/−/^NIC, NIC and WT mice. **D,** pS6 expression was quantified using three randomly selected IHC samples from STK^−/−/^NIC, NIC and WT mice. Data is represented as relative pS6 expression mean ± SEM, p<0.0001. **E**, Carmine alum staining of mammary gland wholemounts from STK11^−/−^NIC (top panel, 2× and 10× magnification) and NIC mice (bottom panel, 2× and 10× magnification). Data are representative of three separate experiments, using three separate mice from each genotype.

Given our results from analysis of human TMAs that indicate 31% of HER2 positive breast cancers lack LKB1 expression (Table 1, Fig. 1), we investigated whether the loss of LKB1 expression would alter the latency of ErbB2-mediated tumorigenesis. To explore the possibility, we developed a stochastic model of human breast cancer by breeding STK11*^fl/f^* mice (FVB) [21] with mice genetically engineered to express activated Neu/HER2-MMTV-Cre (FVB) under the endogenous *Erbb2* promoter, referred to as NIC mice [20], to generate STK11*^−/−/^*NIC mice. Kaplan-Meir curves show a significant reduction in the latency of tumor onset in nulliparous STK11*^−/−/^*NIC mice (Fig. 2A) compared to NIC mice (p<0.0001). By 147 days, 50% of STK11*^−/−/^*NIC mice presented with tumors, compared to NIC mice at 197 days and nulliparous WT (FVB) mice that did not developed tumors (Fig. 2A). Western blot analysis confirmed a significant reduction in LKB1 expression in STK11^−/−/^NIC tumors compared to NIC tumors and WT mammary tissue, and elevated expression of ErbB2 in NIC and STK11^−/−/^NIC tumors (Fig. 2B) as determined by densitometry. Characterization of STK11^−/−/^NIC (top panel) and NIC (middle panel) by IHC confirmed DCIS (Fig. 2C) with loss of LKB1 expression, elevated expression of ErbB2, and pS6, compared to wild-type (bottom panel) mammary glands. Analysis of pS6 expression using imaging software confirmed a significant difference between pS6 expression in STK11^−/−/^NIC tumors compared to NIC tumors (Fig. 2D, p<0.0001), suggesting hyperactive mTOR activity in STK11^−/−/^NIC tumors. We did not observe any difference in tumor histopathology between STK11^−/−/^NIC and NIC tumors. Nor were we able to observed significant metastasis to organs such as lung. Whole-mount analysis of mammary tissue adjacent to primary tumors from STK11*^−/−/^*NIC (top panels) and NIC (bottom panels) (Fig. 2E) mice displayed abnormal luminal growth that likely represent early tumors.

### mTOR and ErbB2 Signaling

**Figure 3:**
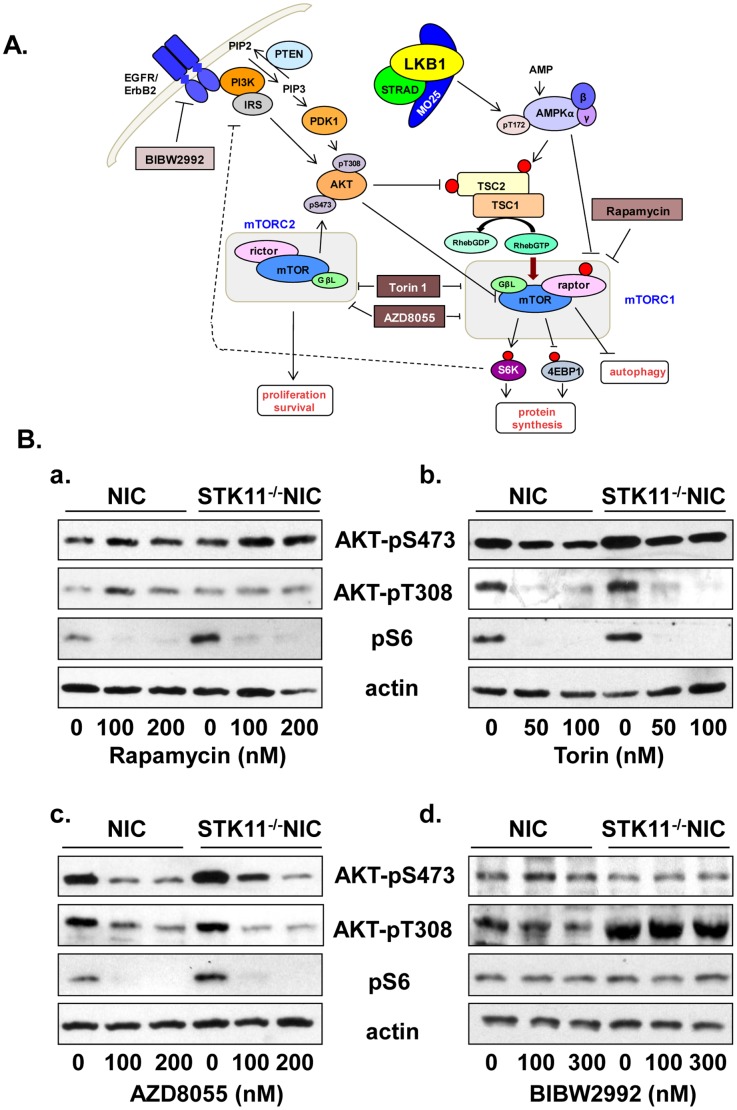
Inhibition of mTOR signaling. **A,** LKB1 in complex with STE20-related adaptor protein (STRAD) and mouse protein 25 (MO25) phosphorylates AMP-dependent protein kinase alpha (AMPKα) on T172. AMPK phosphorylates tuberous sclerosis complex 2 (TSC2) on T1227 and S1345 in response to metabolic stress. Loss of LKB1 expression leads to impaired regulation of AMPK, leading to impaired regulation of mammalian target of rapamycin (mTOR) complex 1 (mTORC1). Rapamycin is an allosteric inhibitor of mTOR complex 1 (mTORC1) that prevents phosphorylation of ribosomal S6 protein (pS6) by S6 kinase 1 (S6K1). Torin1 and AZD8055 are selective ATP-competitive mTOR inhibitors of both mTORC1 and mTOR complex 2 (mTORC2), thereby blocking both protein synthesis and pro-survival pathways mediated by mTOR. With Torin1 and AZD8055, inhibition of mTOR prevents the phosphorylation of S6 by S6K and the phosphorylation of AKT on S473 by mTOR. BIBW2992 irreversibly inhibits the ATP binding pocket within the kinase domain EGFR and ErbB2, whereby phosphorylation of AKT on the conserved T308, via phosphoinositide-3-kinase (PI3K) signaling, is inhibited. PDK1, 3-phospoinositide-dependent kinase 1; PTEN, phosphatase and tensin homologue; eukaryotic initiation factor 4E binding protein 1 (4EBP1); insulin receptor substrate (IRS). **B,** MECs isolated from tumors harvested from NIC and STK11*^−/−/^*NIC mice were treated at concentrations as indicated with the following **a,** Rapamycin for 30 min. **b,** Torin1 for 2 h. **c,** AZD8055 for 2 h and **d,** BIBW2992 for 2 h. Expression of AKT-pS473, AKT-pT308 and pS6 was determined in response to treatments by western blot analysis using antibodies detailed in the Methods section. Data are representative of three separate experiments, using three separate mice from each genotype.

Based on the results described above, we evaluated the expression of downstream targets of mTOR in MECs harvested from STK11*^−/−/^*NIC, and NIC mice, in the absence and presence of mTORC1/mTORC2 and ErbB1/ErbB2 inhibitors (Fig. 3A). In STK11*^−/−/^*NIC mice, phosphorylation of S6, AKT (S473 and T308) were elevated and compared to levels in NIC mice in response to treatment of mTOR with selective inhibitor Rapamycin (Rapa). Here, Rapa attenuated the phosphorylation status of S6 (Fig. 3Ba), while the phosphorylation status of AKT at residues S473 and T308 were unchanged (Fig. 3Ba). Since Rapa is selective for mTORC1, we tested whether inhibition of mTORC1 and mTORC2 by the ATP-competitive inhibitor Torin1 [23] altered mTOR activity. In MECs from STK11*^−/−/^*NIC mice, we observed a significant reduction in the phosphorylation status of pS6 in response to Torin1 in STK11*^−/−/^*NIC, and NIC mice (Fig. 3Bb). Furthermore, we observed inhibition of the phosphorylation status of both pAKT-S473 and pAKT-T308 in response to Torin1. Next we tested AZD8055, a novel ATP-competitive inhibitor of mTOR that inhibits both mTORC1 and mTORC2 complexes thereby preventing feedback loops through AKT [28]. As with Torin1, we observed a reduction in the phosphorylation status of S6 along with inhibition of AKT-pS473 and inhibition of AKT-pT308 (Fig. 3Bc).

To test whether 5- aminoimidazole-4-carboxamide ribonucleoside (AICAR) could restore AMPK activity in the absence of LKB1 and therefore inhibit mTOR activity, we treated MECs from STK11^−/−^NIC with AICAR (up to 2 mM for 1 h) followed by western blot analysis for protein expression. As previously shown by others [11], [25], [29] AICAR did not activate AMPK in the absence of LKB1, as we did not observe any changes in the phosphorylation status of ACC or S6, the substrate of S6K (data not shown).

Since mammary tumors from STK11*^−/−/^*NIC mice are positive for elevated ErbB2 expression (Fig. 2B), and 31% of HER2 positive breast cancers show loss of LKB1 expression (Table 1, Fig. 1), we investigated the effect of a next generation small molecule that targets the EGFR (ErbB1) and HER2, BIBW2992 (BIBW), on mTOR signaling. BIBW is an anilino-quinazoline that irreversibly binds to critical residues in the ATP binding pocket within the kinase domain; Cys 773 of EGFR and Cys 805 of HER2, thereby rendering the tyrosine kinases catalytically dead [30]. Compared to tumors from NIC mice, AKTp308 expression was elevated in STK11*^−/−/^*NIC mice (Fig, 3Bd). Treatment of mammary tumors cells from STK11*^−/−/^*NIC and NIC mice with BIBW did not lead to changes in AKT phosphorylation, nor did we observe changes in the phosphorylation status of S6 (Fig. 3Bd).

### NMR Analysis of Glycolysis Metabolites

**Figure 4:**
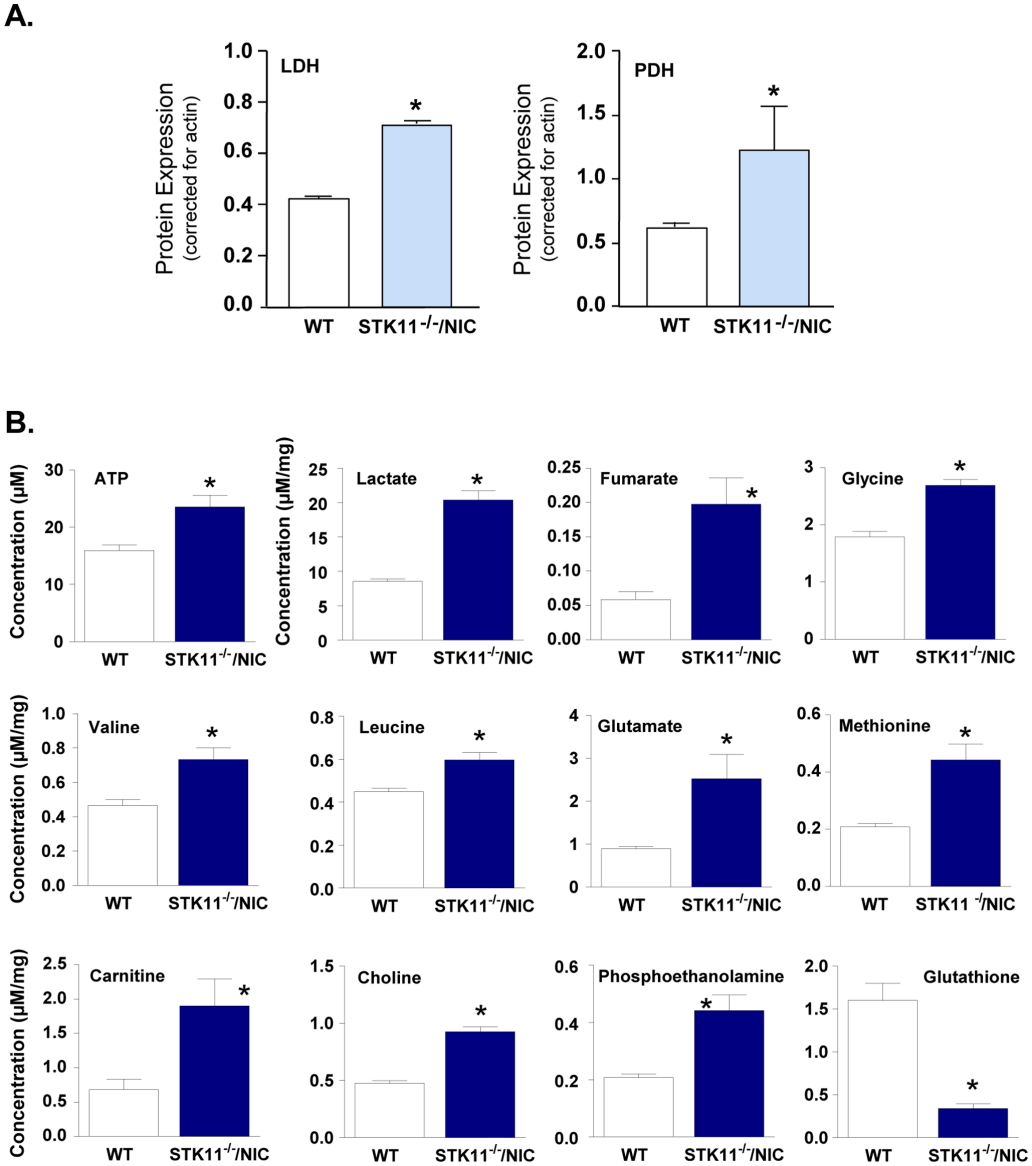
Analysis of metabolites from mammary tumors. Mammary glands from WT mice and tumors from STK11*^−/−/^*NIC mice were excised and analyzed for **A,** expression of LDH, and PDH glycolytic enzymes by western blot analysis. Data are representative of three separate experiments, using three separate mice from each genotype and corrected for actin expression, mean ± SD, p<0.05. **B,** Mammary glands from WT mice and tumors from STK11*^−/−/^*NIC mice were excised and analyzed by NMR. ATP concentrations are represented as mean of values from 2 µg of total protein from MEC lysates ± SEM, p<0.01. NMR data represents the mean of tumors from STK11*^−/−/^*NIC mice and mammary glands form WT mice, corrected for the weight of the individual tumors/tissues and reported as µM/mg ±SEM, p<0.05. Tumors from three separate STK11*^−/−/^*NIC mice and mammary tissue from three separate WT mice were analyzed.

Tumors require changes in metabolism in order to support growth, proliferation and survival as first described by Warburg in the 1920s [31]. The Warburg effect, when cancer cells metabolically remodel by shifting from oxidative phosphorylation to aerobic glycolysis to generate ATP despite the presence of oxygen and being less efficient then TCA [32], [33]. Recent reports in the literature highlight the importance of AMPK/mTOR in glycolysis [25], [34]. Since LKB1 is a regulator of AMPK function [8], [34], we tested whether cell metabolism contributed to mammary gland tumorigenesis in our model. First we found that the expression of LDH and PDH in STK11^−/−/^NIC whole mammary tumors were elevated compare to wild-type mammary glands (Fig. 4A). Next, we determined whether ATP levels were different between normal mammary tissues and mammary tumors by bioluminescence assay. Here we found that ATP levels in primary cells prepared from STK11*^−/−/^*NIC tumors was significantly increased compared to levels in primary cells from WT mammary glands (23.5±2.0 µM and 15.9±0.9 µM, p<0.01 respectively) (Fig. 4B).

Next, we measured metabolites in tumors by NMR and found that lactate was significantly elevated in STK11*^−/−/^*NIC tumors compared to WT mammary glands (20.4±2.2 µM/mg and 8.5±0.5 µM/mg, respectively; p<0.05). Since elevated lactate production is a hallmark of cancer metabolism [35], our results suggest that lactate from tumors could then be used by adjacent epithelial cells to generate ATP through TCA cycle as would fumarate, of which is significantly elevated in STK11*^−/−/^*NIC tumors compared to levels in WT mammary glands (0.19±0.03 and 0.06±0.01 µM/mg, respectively; p<0.05). In addition, we observed elevated levels of glycine in STK11*^−/−/^*NIC tumors compared to WT mammary glands (1.8±0.09 and 2.7±0.1 µM/mg, respectively; p<0.05), supporting the biosynthetic requirements of the growing tumor.

Compared to WT mammary glands, branched-chain amino acids (BCAA) were significantly elevated in STK11*^−/−/^*NIC mammary tumors, specifically valine and leucine. BCAAs are used for translation and are preferentially metabolized by cancer cells to serve as nitrogen sources for the production of the non-essential fatty acid glutamate, a nutrient required by cancer cells in addition to glucose. Glutamate can then be converted to the oxidative substrate α-ketoglutarate and/or converted to pyruvate, both of which are then used in TCA. Compared to WT mammary tissue, glutamate was significantly elevated in STK11*^−/−/^*NIC tumors (p<0.05).

During translation, the essential amino acid methionine is incorporated into the N-terminal position of all proteins as well as serving as an intermediate for biosynthetic products including membrane phospholipids. In STK11*^−/−/^*NIC tumors, we observe an increase in methionine, compared to WT tissues (0.44±0.15 and 0.21±0.01 µM/mg, respectively; p<0.05). Carnitine was significantly elevated in STK11*^−/−/^*NIC tumors, compared to WT tissues (1.9±0.0.7 and 0.68±0.3 µM/mg, respectively; p<0.05), thereby serving as a precursor for mitochondrial ATP production through β-oxidation of fatty acids, satiating the energy requirement of growing tumors [32], [33]. The dysregulation of lipid metabolism in tumors [36] as reflected by significantly elevated levels of choline (0.92±0.04 µM/mg), and phosphoethanolamine (0.44±0.05 µM/mg), compared to levels in WT mammary tissues (0.47±0.02 and 0.20±0.01 µM/mg, respectively; p<0.05). In addition, we observed a reduction in redox metabolism as reflected by the antioxidant enzyme glutathione in STK11*^−/−/^*NIC tumors, compared to WT tissues (1.6±0.2 µM/mg and 0.33±0.06 µM/mg, respectively; p<0.05), thereby providing a micro-environment that is beneficial to the development of the tumors.

### Inhibition of mTOR Induces Alteration in Glycolytic Enzymes

In our model, we observed that in the absence of LKB1 expression, mTOR is hyperactivated thereby enhancing glycolytic enzymes (Fig. 4). Since STK11^−/−/^NIC mammary tumors exhibit a high concentration of lactate secretion (Fig. 4B), a characteristic of the Warburg effect, we investigated whether inhibition of mTORC1 and mTORC2 complexes would translate into changes in lactate dehydrogenase (LDH) expression, as well as pyruvate dehydrogenase (PDH).

**Figure 5:**
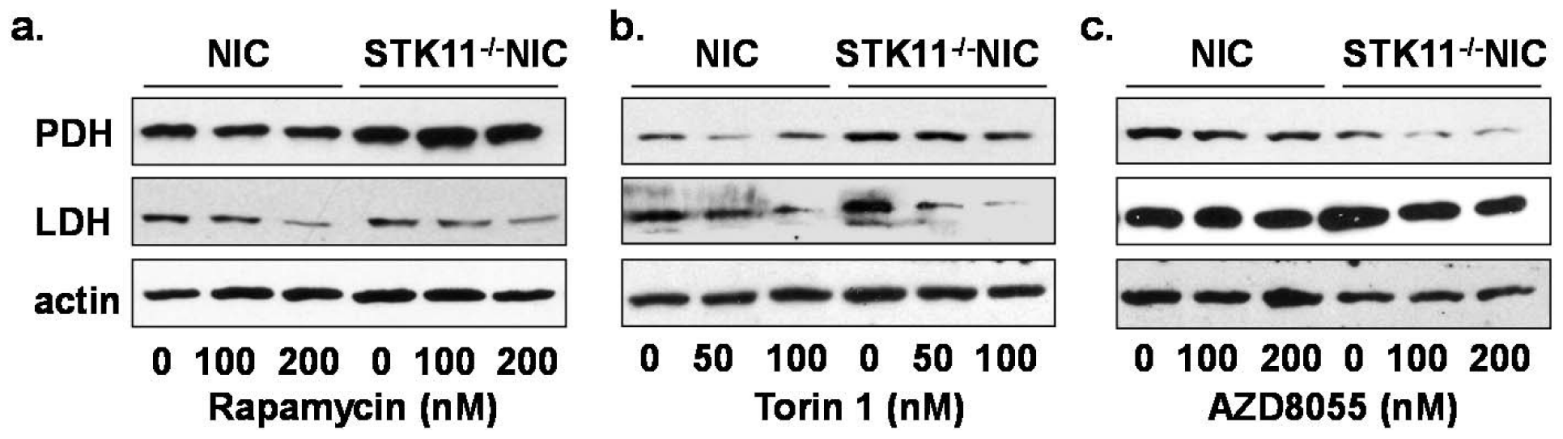
Expression of glycolytic enzymes in mammary tumors. MECs were isolated from STK11*^−/−/^*NIC tumors and NIC mammary glands, followed by treatment with **a,** Rapamycin for 30 min. **b,** Torin 1 for 2 h and **c,** AZD8055 for 2 h, followed by western blot analysis for LDH, and PDH. Data are representative of three separate experiments, using three separate mice from each genotype.

We observed that Rapamycin treatment led to modest reduction in LDH expression in STK11^−/−/^NIC cells compared to NIC however, we did not observe changes in PDH expression (Fig. 5a). Previous work by others show that Rapamycin inhibits lactate secretion in mTORC1 hyperactive cells [33]. Next, we investigated whether treatment of primary tumor cells with Torin1 and AZD would alter the expression of these enzymes. PDH expression was not altered in response to Torin1 treatment in STK11^−/−/^NIC compared to NIC MECs (Fig. 5b) however, PDH expression was modestly reduced in response to AZD (Fig. 5c). In response to both Torin 1 and AZD treatments (Fig. 5b, c) we observed a reduction in LDH expression in STK11^−/−/^NIC compared to NIC MECs. These data suggest that the hyperactivation of mTOR through loss of LKB1 expression in mammary tumors contributes to LDH expression.

## Discussion

Breast cancer is an intricate disease due to the complex interplay between numerous proteins and genes at the molecular level. Because of the stochastic nature of this disease, characterization of critical signaling events that lead to the development and progression of breast cancer require further exploration. Since little is understood about the complex interplay between LKB1 and oncogenes in breast cancer, we evaluated the expression of LKB1 in human breast cancer tissue microarrays and found that 31% of HER2 positive highly invasive cancers were null for LKB1 expression. These data along with studies conducted by others that report reduced expression of LKB1 is correlated with poor prognosis [3], strongly support a role for LKB1 in breast cancer and suggest molecular interplay between LKB1 and HER2 signaling pathways in breast cancer.

In an effort to understand the molecular interplay between LKB1 and HER2, we developed a mouse model where *lkb1* was conditionally inactivated in combination with conditional expression of activated ErbB2 and Cre recombinase in the same mammary epithelial cell [20]. We observed a significant reduction in the latency of mammary gland tumor onset in STK11*^−/−/^*NIC mice compared to NIC mice. We attribute the reduced latency to hyperactivation of mTOR signalling network in response to the loss of LKB1 expression (Fig. 2 and 3). Since LKB1 is a regulator of mTOR activity through the phosphorylation of the energy sensor AMPK, in the absence of this regulation, mTOR is active whereby it phosphorylates S6K1, that in turn phosphorylates pS6, ultimately leading to protein translation [8]. Interestingly, previous work by others show that PTEN^−/−/^NIC mice rapidly developed mammary gland tumors with corresponding amplification of the PI3K/AKT pathway however, mTOR signaling was determined not to be a contributing factor in tumorigenesis [20]. More recently, others have shown that breast cancer cell lines with PTEN loss of function, were not sensitive to second generation mTOR inhibitors, specifically PP242 [37]. Overall, our data suggest that hyperactivation of mTOR signaling in response to loss of LKB1 function and gain of ErbB2 oncogenic activity leading to tumorigenesis may be mechanistically different from PTEN/ErbB2-mediated mammary gland tumorigenesis.

We investigated specific inhibitors of mTOR and ErbB2 signaling pathways (Fig. 3A) whereby Rapa treatment was effective at inhibiting mTOR, however Rapa did not inhibit the feedback loop through S6 kinase as we did not observe inhibition of AKTp473. These data are consistent with the finding from the literature that highlights the Rapamycin-resistant function of mTORC1 [23]. We did not observe inhibition of pS6 or pAKT in response to BIBW2992 treatment, suggestive that inhibition of ErbB2 was not sufficient to inhibit mTOR signaling. However, the ATP-competitive inhibitors of mTORC1-mTORC2, Torin 1 and AZD8055, inhibited pS6 and AKT (pS473 and pT308), proving to be more efficacious at inhibiting mTOR in our model.

Previous work by others have explored the effects of inhibiting mTOR and MAPK signalling and found that Torin 1 [23] and AZD8055 [26], [28] treatment of primary cells, cell lines and xenografts inhibit mTORC1/mTORC2 (mTOR), as well as expression of cyclin D1, and cyclin D3, while increasing expression of the cell cycle inhibitor p27Kip1 [23], [26], [28]. In PTEN/LKB1 deficient mice that develop B-cell follicular lymphoma, AZD8055 treatment resulted in the inhibition of the phosphorylation of AKT substrates PRAS40 and FOXO1/3a, as well as the N-myc downstream regulated gene1 (NDRG1) [26]. Others have shown similar results in several cells lines including the more common MCF7, HEK293, and A549 cells [28]. More recently, AZD8055 treatment of Calu-6 xenografts were found to inhibit mTOR signaling [38] similar to findings from our own study. Furthermore, the authors showed that AZD8055 treatment increased cleaved PARP as well as led to a modest increase in BIM-extra long (BIM-EL), the pro-apoptotic BH3 Bcl-2 family member [38]. In this study, combination treatment with AZD8055 and the MEK inhibitor, AZD6244 (selumetinib), proved to be more efficacious at cleaving PARP and increasing BIM-EL, then AZD8055 alone. Given the recent work by others, pathways identified in their studies may be of interest in our model.

Metabolites are the final product of the genome and are therefore reflective of mutations, deletions, epigenetic or transcriptional modification and are defined as the total quantitative collection of small molecular weight compounds. We observed a difference in the ATP levels between STK11*^−/−/^*NIC mammary tumors and WT mammary tissue, reflective of the loss of LKB1 expression and therefore regulation of AMPK activity, leading to hyperactivation of mTOR through both mTORC1 and mTORC2 pathways. Previous work by others have demonstrated that activation of mTORC1 is sufficient to stimulate specific metabolic pathways that include glycolysis, the oxidative arm of the pentose phosphate pathway, and *de novo* lipid biosynthesis [33]. Metabolic adaptation of STK11*^−/−/^*NIC tumors is consistent with defects in fatty acid oxidation, protein synthesis and redox potential (Fig. 4). Therapies that target multiple metabolic products in combination with aberrant signaling pathways, such LKB1-AMPK-mTORC1 and ErbB2-AKT-mTORC2 may be a consideration for the future of targeted cancer treatments, particularly for HER2 resistant trastuzumab-refractory breast cancer [19]. Currently, there are significant efforts to reverse the Warburg effect through AMPK agonists that would re-establish the conduit between energy and growth signaling to shut down tumor growth. The most common of these agonists are the anti-diabetic drugs metformin and phenformin. It remains to be seen, whether these drugs will be useful in the treatment of cancers exhibiting enhanced mTOR activity resulting from deregulation of multiple signaling pathways that include LKB1.

Recent studies have shown that metabolism and increased glycolysis are two important processes responsible for inducing and sustaining the malignant transformation of normal cells [33], [35]. Previous work by others has shown that treatment of cells with Rapa decreases the expression of LDH, PDH and hexokinase II [33]. In our model, we observe enhanced expression of LDH and PDH (Fig. 4A) and corresponding elevated lactate production (Fig. 4B), allowing for cancer cells to maintain glycolytic metabolism and contribute to the increased acidification of the microenvironment which benefit cancer cells over normal cells [39]. In agreement with our findings, others have shown that hyperactivated mTOR leads to elevated expression of LDH [40]. As such, targeted inhibition of LDH metabolic enzyme may serve as therapeutic targets to block cancer cell metabolism. Torin1 and AZD8055 both reduced the expression of glycolytic enzymes LDH and to a lesser extent PDH from primary tumor cells (Fig. 5c and d). In our model these data suggest that loss of the LKB1/AMPK regulation of mTOR is a strong driver of aerobic glycolysis.

Overall, our discovery confirms that loss of LKB1 expression leads to the development of breast cancer and accelerated ErbB2-mediated oncogenesis. The outcome of our study suggests that the loss of LKB1 expression in HER2 positive breast cancer may serve as a marker for hyperactivation of mTOR, warranting further investigation into combinatorial therapeutics that target LKB1-AMPK-mTOR and glycolytic pathways.

## Supporting Information

Figure S1 **LKB1 antibody conditions for immunohistochemistry.** Human breast cancer tissue microarrays of invasive ductal carcinoma (Biomax USA) were used to establish the concentration of anti-LKB1 antibody for staining by IHC. Left panel represents H/E staining, central panel represents anti-LKB1 staining; **a,** LKB1++ (medium expression), **b,** LKB1+++ (high expression), **c,** LKB1neg (null expression) and **d,** LKB1+ (modest expression), and right panel represents antibody control.
(TIF)
