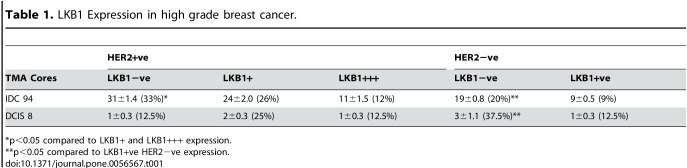# Correction: Loss of *lkb1* Expression Reduces the Latency of ErbB2-Mediated Mammary Gland Tumorigenesis, Promoting Changes in Metabolic Pathways

**DOI:** 10.1371/annotation/f4149a95-c6e6-45f6-9a6a-2d4f48f8d62c

**Published:** 2013-02-26

**Authors:** Rafaela Andrade-Vieira, Zhaolin Xu, Patricia Colp, Paola A. Marignani

In Table 1, the first entries under the headings "TMA Cores" and "LKB1-ve" were incorrect. The first entry under the heading "TMA Cores" should read "IDC 94". The first entry under the heading "LKB1-ve" should read "31±1.4 (33%)*". The following footnotes were omitted from Table 1: "*p<0.05 compared to LKB1+ and LKB1+++ expression" "**p<0.05 compared to LKB1+ve HER2-ve expression". Please see the corrected Table 1 here: 

**Figure:**