# Supplementary figures and images for: Loss of lkb1 Expression Reduces the Latency of ErbB2-Mediated Mammary Gland Tumorigenesis, Promoting Changes in Metabolic Pathways
Source: PLoS One. 2013 Feb 22;8(2):e56567. doi: 10.1371/journal.pone.0056567 (PMC3579833; doi:10.1371/journal.pone.0056567)

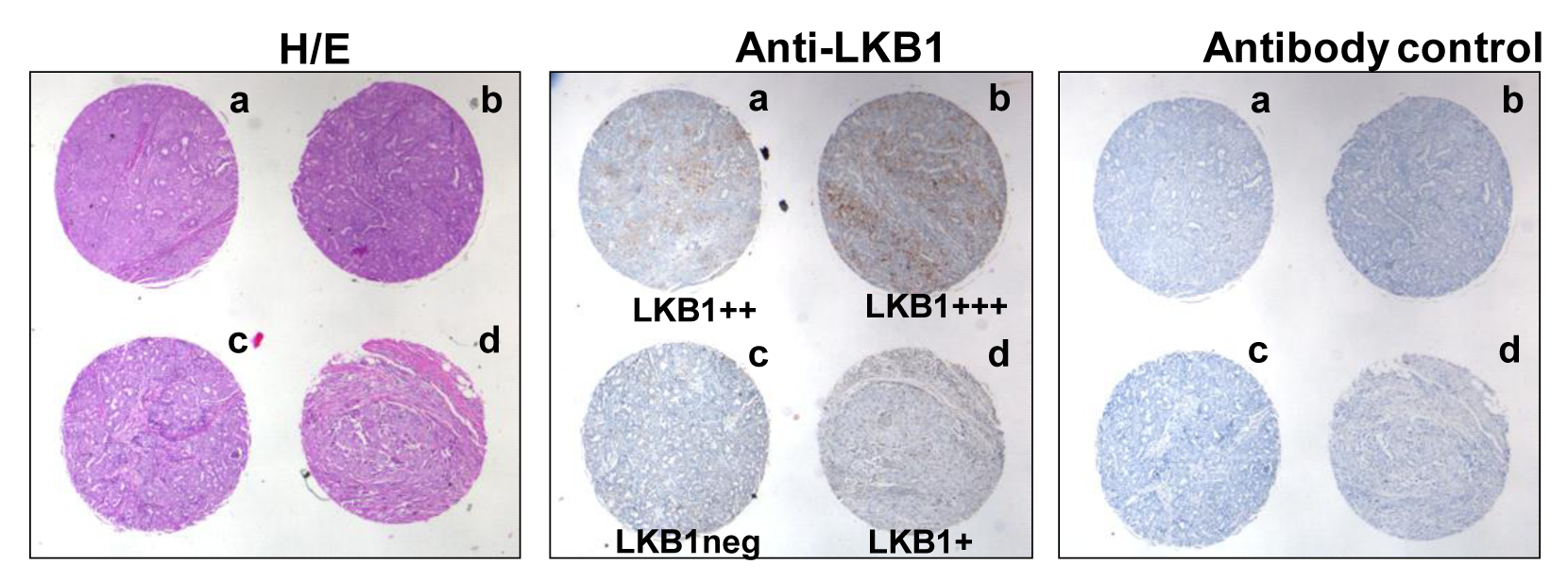

Supplement: Figure S1 — LKB1 antibody conditions for immunohistochemistry. Human breast cancer tissue microarrays of invasive ductal carcinoma (Biomax USA) were used to establish the concentration of anti-LKB1 antibody for staining by IHC. Left panel represents H/E staining, central panel represents anti-LKB1 staining; a, LKB1++ (medium expression), b, LKB1+++ (high expression), c, LKB1neg (null expression) and d, LKB1+ (modest expression), and right panel represents antibody control. (TIF) [file pone.0056567.s001.tif]
